# International needs analysis in orthopaedic trauma for practising surgeons with a 3-year review of resulting actions

**DOI:** 10.1080/21614083.2017.1398555

**Published:** 2017-11-15

**Authors:** Richard Buckley, Peter Brink, Kodi Kojima, Wa’el Taha, Donald Moore, Mike Cunningham, AOTrauma Global Needs Analysis Group

**Affiliations:** ^a^ Foothills Medical Center, University of Calgary, Alberta, Canada; ^b^ Department of Traumatology, Maastricht University Medical Center, Maastricht, The Netherlands; ^c^ Hospital das Clinicas HCFMUSP, Faculdade de Medicina Universidade de Sao Paulo, Sao Paulo, Brazil; ^d^ King Abdulaziz Medical City, Al-Madinah, Saudi Arabia; ^e^ Office for Continuing Professional Development, Vanderbilt University Medical Center, Nashville TN, USA; ^f^ AO Foundation, AO Education Institute, Duebendorf, Switzerland

**Keywords:** Surgeon education, orthopaedic trauma, needs analysis, needs assessment, curriculum planning, educational gaps, educational preferences

## Abstract

**Introduction**: To ensure best-quality education in orthopaedic trauma, the AOTrauma Education Commission conducted a Global Needs Analysis with practising surgeons worldwide.

**Material and methods**: During July to November 2012, an email invitation to complete an online set of 30 questions in eight languages was sent to our members and associates in all countries through AOTrauma’s regional networks. Non-members were invited to participate through collaboration with orthopaedic societies.

**Results**: A total of 3,790 surgeons practising orthopaedic trauma (49%), orthopaedic (15%), general trauma (15%) and specialty orthopaedic (13%) surgeons responded worldwide. Seventy per cent completed all questions, and the top 10 countries accounted for half the responses. The top 3 areas of educational need were orthopaedic trauma, joint replacement and preservation, and pelvis and acetabulum. Aspects influencing likelihood to attend face-to-face courses were: expert faculty, focus on a specific topic, clear objectives, and discussion and feedback from experts. Barriers to attending courses were time away from practice, cost and lack of availability or access.

**Conclusion:** The Global Needs Analysis helped our educational committees to identify short- and mid-term priorities over recent years. Adjustments in our planning have helped meet the needs of our audience on a global, regional and national level.

## Introduction

The AO Foundation is a medically guided non-profit organisation led by an international group of surgeons specialised in the treatment of trauma and disorders of the musculoskeletal system. To enhance patient care worldwide, one of the missions of the organisation has been to foster education among health care professionals. Having been involved in this educational endeavour since its inception in 1958, the organisation had a strong mandate to determine a more responsive vision to the continuing professional development (CPD) requirements of its worldwide surgeon base.

Throughout lifelong learning, a practising surgeon may become out of date and develop educational gaps because of new technology and techniques, practice pattern change or lack of access to education [1]. Performance gaps can also develop gradually over time, often as several smaller specific knowledge or skills gaps that can be effectively addressed by an educational intervention [2]. CPD is the mechanism by which doctors keep their practice up to date and is defined as “any and all ways by which doctors learn after formal completion of their training”, and its primary purpose is to maintain and improve clinical performance [3].

In 2011, CPD within AOTrauma (a surgeon specialty group focused on orthopaedic trauma within the AO Foundation) was characterised by courses for multiple surgeon learner levels, but educational activities, especially for senior surgeons, were sometimes unstructured and inconsistent in terms of quality. AO educational planning had responded to evaluations from previous courses, but a cohesive, centrally developed vision with in-depth knowledge regarding the needs of the surgeon learner was not available. What could future, improved AOTrauma CPD look like? How could it be optimally designed to meet gaps in professional practice, knowledge, skills and attitudes? We needed a strong international initiative to identify needs to help improve our educational offerings to practising surgeons.

AOTrauma decided to gather comprehensive needs assessment data to identify educational gaps within their worldwide target audience and to gain better insight into practice profiles, patterns and settings. This project would provide important data for backwards planning and would set the stage for programme design decisions, set standards and markers, and provide baseline information to measure if gaps had been met. The target audiences were identified as practising (qualified/board certified) surgeons at the beginning of practice, in various levels of growing specialisation, and experienced and expert surgeons in all types of hospital settings. The needs analysis would provide a thorough understanding of the needs of our worldwide target audience and any variations at regional and national levels. It would help AOTrauma understand the audiences’ attitudes towards education, level of competency and gaps, clinical problems that are a challenge for them, learning patterns and preferences, work environment and how they integrate learning into their practice.

A strong committee structure for development of change within an organisation exists within AOTrauma, including regional committees, subspecialty curriculum taskforces, educational working groups and CPD coordinators to bring input, implementation, ideas and feedback to an international education commission for consideration and dissemination. With these defined international frameworks, roles and processes, the aim of this initiative was to create goal setting for CPD improvements in all regions and subspecialties of orthopaedic trauma with the use of a carefully planned global needs analysis. Specific questions to be answered by the project were:

1. In what specialty and subspecialty areas do orthopaedic trauma surgeons need education?
2. How do they want to receive education to meet their needs?
3. Are there any differences in the needs and preferences in the regions and in specific countries?
4. What barriers do surgeons have in accessing the education they need?

## Methods

**Figure 1.**
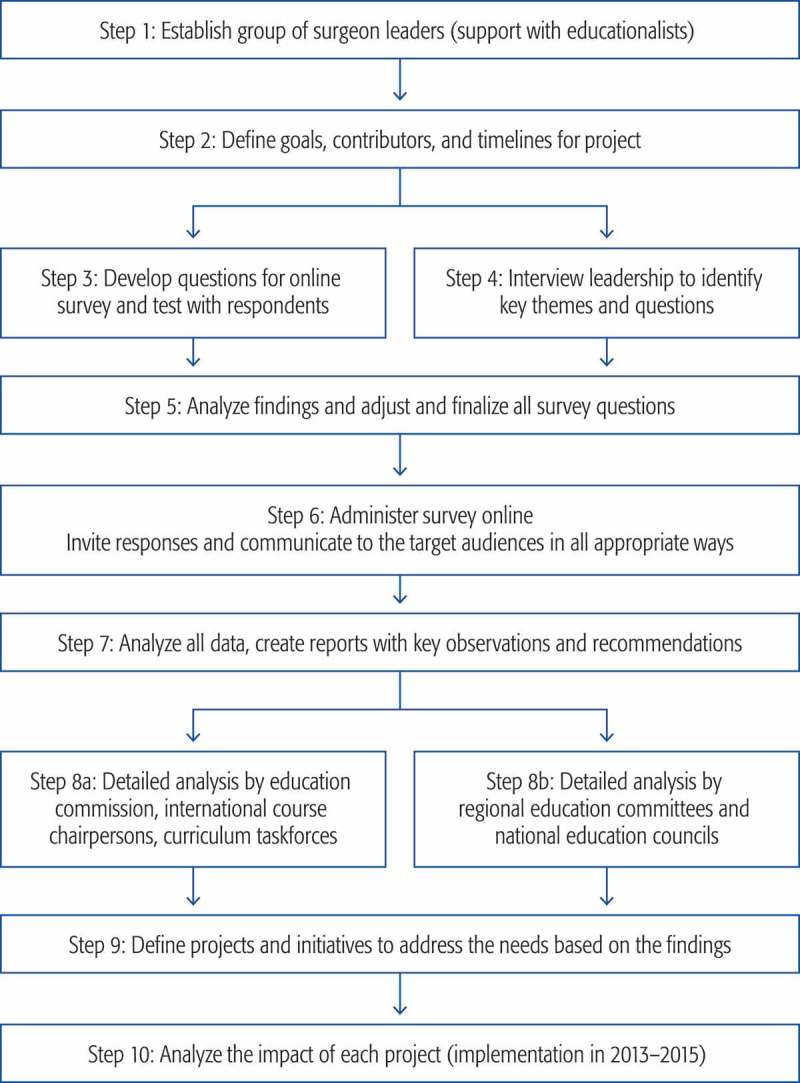
Steps in AOTrauma global needs analysis (mixed methods approach).

A mixed methods needs analysis project was approved and started in November 2011 [4]. The three main sources of information to determine the needs and barriers were: (1) an advisory panel of expert surgeons, (2) online responses from practising surgeons worldwide and (3) structured interviews with AOTrauma’s surgeon leaders (international board, education commission and AO Foundation leaders). The 10 steps are outlined in Figure 1 and were completed over a period of 2 years.

1. A face-to-face meeting was held with three surgeon experts and international faculty and three educationalists. A small group of other key surgeons were interviewed to provide input and guidance.
2. To gain a thorough understanding of our CPD audience and their needs, the group decided to conduct an online needs assessment worldwide and to interview surgeon leaders from all regions. An ethics application submitted to Maastricht University was approved with exemption [METC 12–5-054].
3. A set of 23 questions on practice profile, educational needs, preferred educational formats and barriers to participation in face-to-face courses was designed for pilot testing. Two pilot sites were identified in Alberta, Canada and Limburg, The Netherlands, and 52 responses were gathered and analysed.
4. A leading external expert in CME conducted 20 thirty-minute face-to-face and online/phone interviews with our surgeon leaders using a structured set of eight questions.
5. Based on the responses to the pilot questions and key findings from the interviews, the project committee met and adjusted the questions to create a final set of 30 items. These were translated into eight languages by professional translators and approved by local surgeons.
6. An email campaign to the entire AOTrauma network (approximately 20,000 practising surgeons worldwide) was created, region by region. In order to reach a reasonable proportion of non-members or associates, invitations were also sent through collaboration with many national and regional orthopaedic societies.
7. All data were gathered using SurveyMonkey and analysed using Microsoft Excel. Visualisation of the key data charts was completed using Tableau and InDesign programmes. Reports were generated in hard copy and printable electronic formats. The expert surgeons in the project committee identified key observations and recommended the next steps for all regions.
8. The reports were circulated to each region, to the top 10 countries in response rates and to all AOTrauma educational taskforces responsible for the various subspecialty areas. Each group reviewed the data during one of their national or education-focused meetings.
9. The regional education committees presented their planned actions based on the data at the international education commission meeting in October 2013. Educational taskforces reviewed the data during their curriculum meetings and used the information to help guide their planning decisions.
10. During the 3 years following the needs-assessment project, the data were used by surgeons in committee and taskforces to help decide on the topics and content for the annual AOTrauma Davos courses, to select topics for 1-day seminars and to identify content for webinars, apps and online modules.

## Results

A total of 4,316 responses were gathered online between July and October 2012, with 70% completing all 30 questions. All regions were well represented, and the top 10 countries accounted for approximately half the responses. Responses from residents and non-surgeons were separated, leaving an overall dataset of 3,790 practising surgeons (breakdown and profiles as well as reported reasons for actively taking part in educational activities are shown in Table 1).

**Table 1.** Profile of responders to AOTrauma’s global needs analysis by region (Global = all regions).

	Percentages for regions and globally					
Question	Europe	North America	Middle East	Asia Pacific	Latin America	Global
Current position (practising surgeon doing mostly …)						
Orthopaedic trauma	42	54	63	59	42	49
General orthopaedics (joint replacement, etc.)	12	20	16	21	14	15
General trauma	25	1	5	4	12	14
Specialty orthopaedics	12	18	10	11	22	13
Percentage of time on trauma						
0–30	30	40	27	39	28	32
40–60	36	23	52	43	46	40
70–100	34	37	21	19	26	28
Current stage in career						
Start of practice	20	17	20	17	14	18
Growing specialisation	38	25	40	48	36	40
Expertise	42	58	40	35	50	42
Graduated from medical school						
1992 and earlier	39	48	45	31	37	38
1993–1997	17	12	16	19	16	17
1998–2002	20	21	15	27	23	22
2003–2007	23	19	24	23	24	23
Fracture surgeries per week						
1–5	47	34	37	42	46	44
6–10	39	40	40	41	41	40
11–15	10	19	17	13	9	11
More than 15	4	8	7	4	4	5
Main practice setting						
Level I trauma centre	37	64	31	39	36	38
Level II trauma centre	30	16	35	31	28	29
Local or community hospital	30	15	19	18	23	24
Private practice	4	5	15	12	13	9
Reasons for taking part in educational activities: strongly disagree = 1, strongly agree = 5 (values <4 are shown in italics)						
When I encounter new patient care challenges or problems	4.35	4.47	4.17	4.44	4.63	4.41
To keep up to date in general in the area of orthopaedic trauma	4.23	4.34	4.33	4.47	4.60	4.38
To meet CME or other regulatory requirements	*3.07*	*3.59*	*3.37*	*3.24*	*3.39*	*3.22*
To learn about state-of-the art treatment options and the latest concepts and technology	4.38	4.12	4.47	4.50	4.67	4.47
To focus on a specific topic or area of practice	4.09	4.07	*3.88*	4.22	4.29	4.14
To improve operative techniques	4.48	4.41	4.60	4.59	4.73	4.57
To improve decision making	4.40	4.31	4.55	4.61	4.69	4.52
To improve preoperative assessment and diagnosis	4.27	4.07	4.30	4.46	4.62	4.39
To improve treatment selection and planning	4.42	4.24	4.40	4.56	4.72	4.51
To improve ability to recognise and manage complications	4.39	4.26	4.42	4.50	4.71	4.48

**Figure 2.**
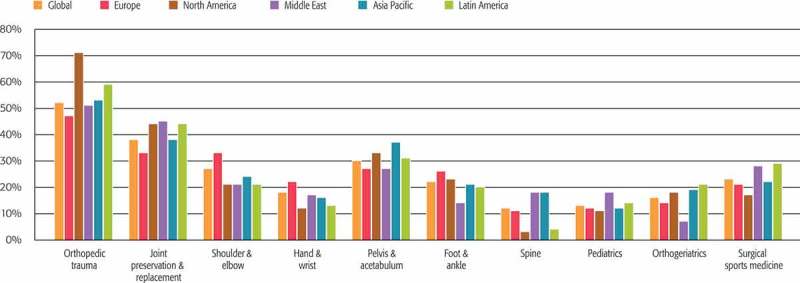
Areas for which responders reported being most likely to seek education during the next 2 to 3 years (% “yes” responses for regions and topics).

The top 3 areas of educational need were orthopaedic trauma (52% of responders), joint replacement and preservation (38%), and pelvis and acetabulum (30%) (Figure 2 and Table 2). The need for the other subspecialty areas ranged from 27% down to 12%, with some variability depending on the region.

**Table 2.** Ranking of categorised responses from 10 countries with the most responders globally.

	Country									
Question	Russia	Brazil	India	Germany	China	Japan	USA	Spain	UK	Switzerland
Areas of educational need in the next 2–3 years (up to 3 allowed per responder)										
Orthopaedic trauma	4	1	2	1	1	1	1	1	1	1
Joint preservation and replacement	3	2	1	2	3	4	2	3	2	4
Shoulder and elbow	2	7	5	4	3	6	5	3	4	2
Hand and wrist	6	9	10	8	9	3	9	5	7	6
Pelvis and acetabulum	1	3	3	5	2	2	3	2	9	8
Foot and ankle	5	6	7	7	5	5	4	9	5	4
Spine	9	10	6	3	6	10	10	8	10	10
Paediatrics	10	8	8	9	10	8	8	10	7	9
Orthogeriatrics	8	5	9	10	7	7	6	6	5	6
Surgical sports medicine	7	4	4	6	8	9	7	7	3	3
Preferred ways to receive education										
Face-to-face events										
Local hospital meetings	11	6	11	6	4	11	7	7	6	7
Meetings/congresses of societies	5	5	6	4	3	2	1	8	2	4
Courses (including AO)	1	1	1	2	5	1	2	1	1	3
Courses delivered by industry	9	7	8	10	11	6	11	11	8	10
Online and self-directed learning										
Books	3	4	4	3	1	4	5	3	5	6
Journals	4	3	3	1	2	3	3	4	3	1
Webinars and webcasts	8	9	7	9	9	9	6	5	10	9
Online forums/cases	6	11	9	11	7	8	10	9	11	11
Mobile learning	10	8	10	8	10	10	9	10	9	8
Online videos	7	10	5	7	8	7	4	6	7	5
AO Surgery Reference	2	2	2	5	6	5	8	2	4	2
I am more likely to attend a course if it …										
Focuses on specific clinical topic	3	5	3	2	1	1	2	1	6	1
Has clear goals and objectives	4	2	2	1	1	3	3	4	2	2
Is delivered by expert faculty	1	1	1	5	3	2	1	8	1	4
Takes no more than 2 days away from my practice	9	8	6	6	8	7	6	7	8	8
Covers broad/general aspects of orthopaedic trauma	10	9	9	9	9	8	10	10	10	9
Includes networking with peers and faculty	6	7	8	10	6	9	8	5	7	6
Provides opportunity to discuss and get feedback from experts	2	4	4	4	4	4	4	2	4	3
Is delivered locally and in the local language	5	10	10	6	7	5	9	9	9	10
Is commercially unbiased	8	6	5	8	5	10	7	6	5	7
Has competitive fee and costs	7	3	7	3	10	6	5	2	3	5
Reasons why you and your colleagues do not attend more face-to-face courses										
Cost	1	2	2	2	2	4	2	2	2	2
Time away from practice	2	1	1	1	1	6	1	1	1	1
Lack of availability/access	3	3	3	7	3	4	4	3	3	4
Content/format issues, language	5	4	4	3	4	1	3	5	4	3
Miscellaneous	7	5	4	4	4	3	5	4	5	4
Lack of publicity or organisation	6	6	5	6	7	6	6	6	–	–
Faculty-related issues	–	7	5	4	4	6	6	7	–	–
Lack of incentive or interest	7	7	3	7	8	–	–	6	–	–
Too many courses/competition	1	7	2	7	–	–	8	8	–	–

When asked about their current use of various educational formats and how they would prefer to receive education in the future (both CME accredited and non-accredited), the formats with ratings over 4.0 out of a possible 5 were (in descending order): courses (including AO), journals, AO Surgery Reference, consultation with experts, meetings/congresses of societies and books. The rankings of the various educational formats in the top 10 responding countries are shown in Table 2. The gaps between present and future preferred use were high for several forms of online learning (forums/cases, webinars/webcasts, mobile learning, and videos) as well as for courses.

**Figure 3.**
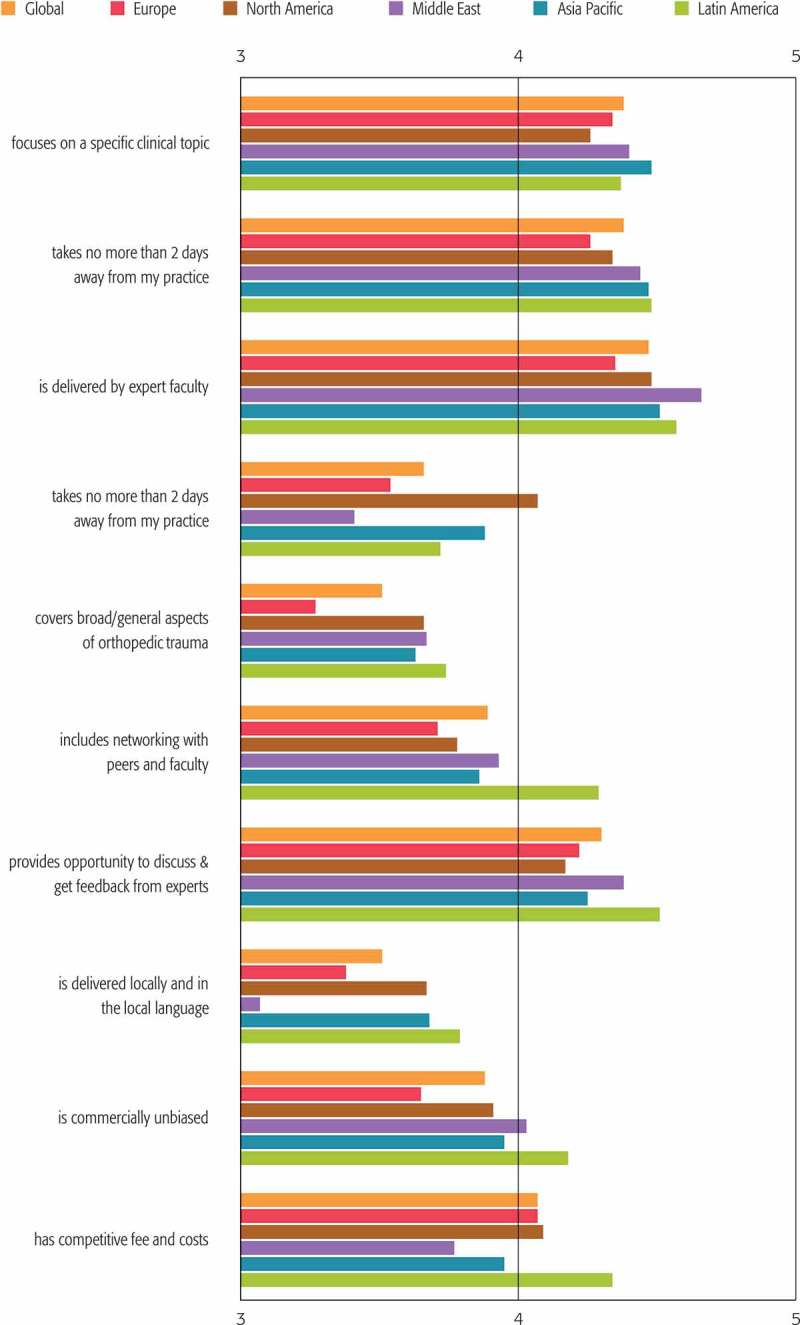
Regional and global responses to question “I am more likely to attend a face-to-face course for practising surgeons if it ….” Level of agreement on each statement: 1 = strongly disagree, 5 = strongly agree.

The aspects reported as most influencing the likelihood of attending face-to-face courses in all regions were: “delivered by expert faculty” (4.47), “focuses on a specific clinical topic” (4.38), “has clear goals and objectives” (4.38) and “provides opportunity for discussion and to get feedback from experts” (4.30) (Figure 3). Barriers to attending courses were time away from practice (rated the number 2 reason in the overall project and the number 1 reason in seven of the top 10 responding countries), cost (rated the number 1 reason overall and rated the number 2 reason in eight of the top 10) and lack of availability or access (rated the number 3 reason overall and in six of the top 10). (Table 2).

## Discussion

Norman et al. stated that “Without a grounding and justification of the content through a specific needs assessment, educational offerings are unlikely to be effective” [5]. Matching content to needs is essential in orthopaedic surgery education, where barriers to putting what has been learned during educational events into practice have been reported [6]. Moore et al. explain how to use the gap-analysis approach to needs assessment when performing backwards planning [7]. This helps education planners recognise where to begin planning learning activities for physicians (a gap analysis should be completed at each outcome level starting with level 7 until no gap is detected). When it comes to the impact of CME on performance and on patient health outcomes, Cervero and Gaines remind us that one of the four primary influences of improved outcomes was that CME is based on practice-based needs assessment [8]. In summary, a deeper insight into practice and practice needs and gaps within a specialty area should be of great benefit for an educational organisation.

The key observations that we extracted from the data (Table 2) were analysed and interpreted by many groups within AOTrauma, and each group identified one or more actions to implement based on the data. AOTrauma’s audience for CPD is a broad range of surgeon types with great variation in their percentage of practice time dedicated to orthopaedic trauma. The AOTrauma Education Commission needs to ensure that our education addresses the needs of three main stages that we define for lifelong learning in practising surgeons: start of practice, growing specialisation and expert surgeon. To ensure every education event is optimised for the participants, the questions used in this needs assessment have been adapted and implemented as an online targeted pre-event assessment of each individual education event. There continues to be a high level of need in all educational areas where we have traditionally focused (orthopaedic trauma and subspecialty fractures). However, there is also a high level of need in several other new areas (joint replacement and preservation, surgical sports medicine, shoulder and elbow). In response, AOTrauma and AO have continued to invest in international curriculum taskforces (planning committees) for the key areas of our education planning and delivery.

Our data show there is a preference for increased use of many educational formats, particularly courses, webinars and other online education, and consultation with experts. The main barriers to participating in face-to-face education are time away from practice, cost and access to, or availability of, courses. We have increased the number of shorter educational events (with a greater focus on specific topics) over recent years and have started to develop more local educational formats (hospital-based education). Because expert faculty are reported as being so important for the likelihood of surgeons attending courses, AO has expanded its faculty development programmes in recent years and now places increased emphasis on implementation of our competency-based education for chairpersons and faculty.

Some regional and national differences were detected in almost all areas of response in the data (e.g. low needs for spine education in our North and Latin America regions, low need for orthogeriatrics in our Middle East region). This information has helped in the planning of international initiatives. However, probably the most important value to our organisation was the ability to provide regional and national reports for more local committees to analyse and plan their yearly educational events (both face to face and online). Differences in generations were detected for the preferred educational formats, with a trend towards increased preference for almost all formats in the younger generations (data on file).

Our reported data add to some recent publications on how to conduct multi-country needs assessments and on the preferences of physicians for various formats of education [9–12]. Direct comparisons are difficult, but there seems to be continued interest and value for both face-to-face and online education.

Strengths of this study include the huge size of this detailed survey and the penetration that AOTrauma has into the fabric of most countries worldwide. AOTrauma, with a carefully organised international administrative structure, has been successful in developing and implementing a very generalisable survey that should be applicable to all adult surgical CPD. The profiling questions from the global needs analysis were refined and now form the focus of a standardised pre-event assessment implemented online before every face-to-face and online educational event enabling ongoing evaluation that is based on the same areas as the needs assessment.

Some of the main limitations of this project were a lack of objective hospital or patient outcomes data to confirm whether the perceived needs match the actual needs from the healthcare system. Also, there was a lack of focus groups with responders to gain deeper insights into the data. In more recent years, our colleagues de Boer and Thorley Wiedler have conducted detailed research on the specific needs of community-based surgeons [13,14]. Future work could investigate these aspects and should also examine if our educational events help meet the needs that were identified by surgeons worldwide. We also need to gather all new information regarding orthopaedic trauma and predicted trends based on changing epidemiology, demographics and training programmes to ensure that AOTrauma continues to meet the evolving needs of surgeons in all countries [15,16].

**Figure 4.**
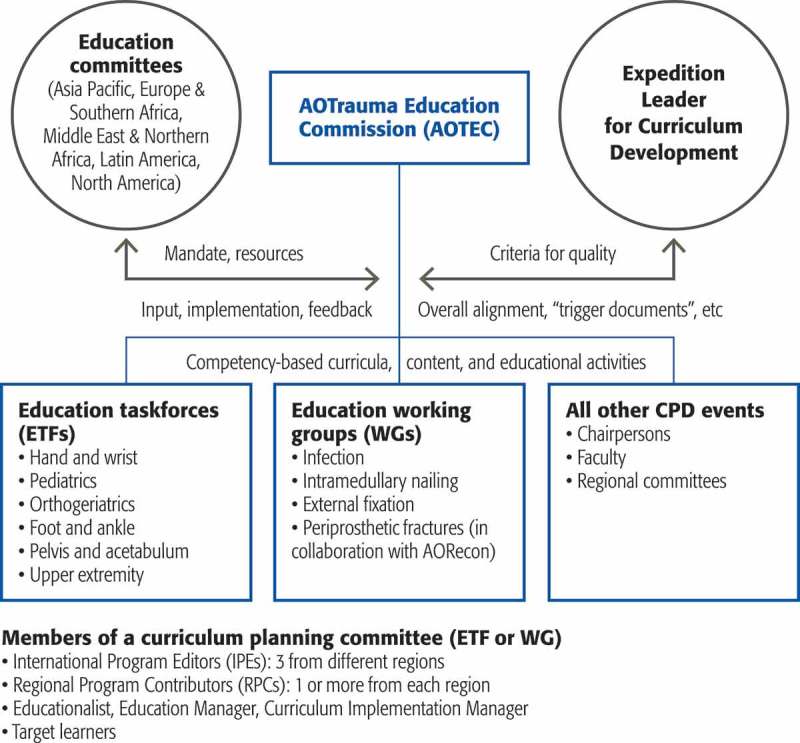
Organisation of continuing professional development (CPD) in AOTrauma.

In 2012, Olson predicted that there would be “continuing challenges in articulating the distinctive role of CPD among all the other entities that are organised to improve practice” [17]. Over the past 3 years, AOTrauma has further evolved its CPD to continue to improve its offerings and to communicate these events to our worldwide audience of practising surgeons (Figure 4) as all groups now develop competency-based education [18]. New educational taskforces and working groups have been added, and these groups of expert surgeons take a leadership position globally to improve education in these areas with the ultimate goal of providing better worldwide patient care. In conclusion, we believe the data from the Global Needs Analysis project have helped many of our regions and curriculum groups and have raised both awareness and the use of assessment data for education planning at all levels.

## AOTrauma Global Needs Analysis Group

Rick Buckley, Kodi Kojima (Chairperson of AOTEC), Wa’el Taha (Expedition Leader for Curriculum Development), Peter Brink, Mark Reilly, Carlos Dominguez Barrios Morales, Wilson Li, and Mahmoud Odat (AOTEC members during the project), Rami Mosheiff, Michael Baumgaertner, Mauricio Kfuri, Chris Morrey, Mamoun Kremli (Regional chairpersons in 2012), Nikolaus Renner (International Chairperson, AOTrauma), Jaime Quintero and Suthorn Bavonratanavech (AO Foundation President and President Elect in 2012), Don Moore, Mike Cunningham, Urs Rüetschi, Clint Miner, Piet de Boer, Jane Thorley Wiedler, Mike Redies, Claude Martin Jr, the AOTrauma Educational Taskforces who reviewed and analysed the data, and the many surgeons and AOTrauma groups who promoted the project in their region and country and who presented the data at regional congresses.

Thanks also to all the team members from the AO Education Institute, AOTrauma, Quantum, and Nougat who helped with setting up, promoting and reporting the data, as well as Tomasso Plebani for his help with the statistical analysis, and all the translators and surgeons who prepared the many versions of the survey worldwide.
